# *catena*-Poly[[trimethyl­tin(IV)]-μ-2-(3-thien­yl)acetato]

**DOI:** 10.1107/S1600536808040762

**Published:** 2008-12-10

**Authors:** Minglei Yang, Handong Yin, Liyuan Wen, Wenkuan Li, Daqi Wang

**Affiliations:** a College of Chemistry and Chemical Engineering, Liaocheng University, Shandong 252059, People’s Republic of China

## Abstract

The title compound, [Sn(CH_3_)_3_(C_6_H_5_O_2_S)]_*n*_, forms an infinite chain structure parallel to [100]. There are two mol­ecules of the complex in the asymmetric unit. The geometry of the Sn atoms in both mol­ecules is distorted trigonal-bipyramidal. The S and C atoms of the thio­phene rings in both mol­ecules are disordered over two sites with site-occupancy factors 0.799 (9)/0.201 (9) and 0.618 (7)/0.382 (7), respectively.

## Related literature

For related structures, see: Addison *et al.* (1984 ▶); Ma *et al.* (2006 ▶).
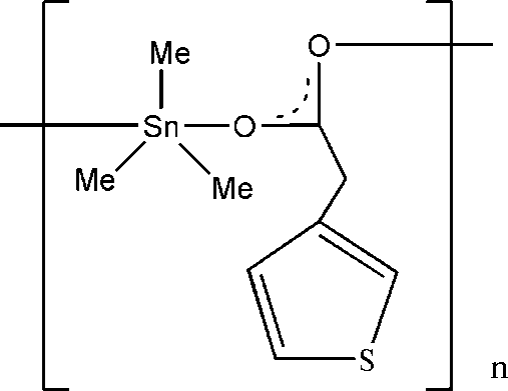


## Experimental

### 

#### Crystal data

[Sn(CH_3_)_3_(C_6_H_5_O_2_S)]
*M* *_r_* = 304.95
Triclinic, 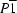

*a* = 10.0677 (9) Å
*b* = 10.839 (1) Å
*c* = 13.2024 (17) Å
α = 107.813 (2)°
β = 105.606 (1)°
γ = 105.030 (1)°
*V* = 1226.0 (2) Å^3^
*Z* = 4
Mo *K*α radiation
μ = 2.23 mm^−1^
*T* = 298 (2) K
0.55 × 0.50 × 0.48 mm

#### Data collection

Bruker SMART CCD area-detector diffractometer
Absorption correction: multi-scan (*SADABS*; Sheldrick, 1996 ▶) *T*_min_ = 0.374, *T*_max_ = 0.415 (expected range = 0.310–0.344)
6334 measured reflections
4227 independent reflections
2865 reflections with *I* > 2σ(*I*)
*R*_int_ = 0.039

#### Refinement

*R*[*F* ^2^ > 2σ(*F* ^2^)] = 0.046
*wR*(*F* ^2^) = 0.143
*S* = 0.97
4227 reflections
282 parameters
H-atom parameters constrained
Δρ_max_ = 0.98 e Å^−3^
Δρ_min_ = −0.94 e Å^−3^

### 

Data collection: *SMART* (Siemens, 1996 ▶); cell refinement: *SAINT* (Siemens, 1996 ▶); data reduction: *SAINT*; program(s) used to solve structure: *SHELXS97* (Sheldrick, 2008 ▶); program(s) used to refine structure: *SHELXL97* (Sheldrick, 2008 ▶); molecular graphics: *SHELXTL* (Sheldrick, 2008 ▶); software used to prepare material for publication: *SHELXTL*.

## Supplementary Material

Crystal structure: contains datablocks I, global. DOI: 10.1107/S1600536808040762/pv2123sup1.cif

Structure factors: contains datablocks I. DOI: 10.1107/S1600536808040762/pv2123Isup2.hkl

Additional supplementary materials:  crystallographic information; 3D view; checkCIF report

## Figures and Tables

**Fig. 1.**
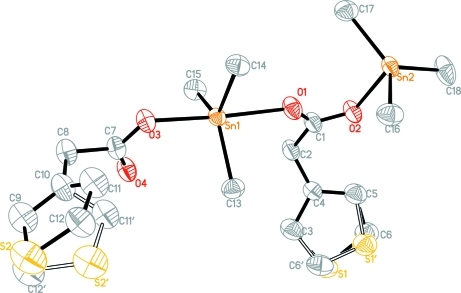
The molecular structure of (I), showing 50% probability displacement ellipsoids and the atom-numbering scheme. H atoms have been omitted for clarity.

**Fig. 2.**
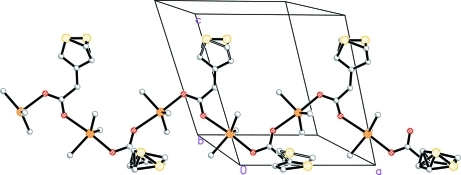
A view of the unit cell showing polymeric chains of (I), H atoms have been omitted for clarity.

## supplementary crystallographic information

### Comment

The title compound (Fig. 1), possesses an infinite one dimensional chain structure arising from Sn—O bridges to the ligand. The Sn1 atom has distorted trigonal-bipyramidal geometry, with atoms O1 and O3 in axial positions [O1—Sn—O3 = 174.7 (2) °] and the C atoms of the three methyl groups in equatorial positions. Associated with the sum of the angles subtended at the Sn1 in the equatorial plane is 359.2 (4) °, indicating approximate coplanarity of these atoms; the Sn1—O1 and Sn1—O3 distance, 2.367 (6) and 2.174 (6) Å, repectively, are close to the corresponding distances reported in organotin compounds (Addison *et al.*, 1984; Ma *et al.*, 2006). The environment of the Sn2 atom is approximate to Sn1.

### Experimental

The reaction was carried out under nitrogen atmosphere. 3-Thiophenemalonic acid (1 mmol) and sodium ethoxide (2.2 mmol) were added to benzene (30 ml) in a Schlenk flask and stirred for 0.5 h. Trimethyltin chloride (2 mmol) was then added and the reaction mixture was stirred for 12 h at 348 K. The resulting clear solution was evaporated under vacuum. The product was crystallized from a mixture of ether/petroleum ether (1:1) to afford the title compound unexpectedly.

### Refinement

The atoms S1, S2, C6, C11 and C12 were found to be disordered over two sites, and the ratio of the occupancy factors refined to 0.680 (7):0.320 (7), 0.624 (7):0.376 (7), 0.799 (9):0.201 (9), 0.624 (7):0.376 (7) and 0.624 (7):0.376 (7) for atoms S1:S1', S2:S2', C6:C6', C11:C11' and C12:C12', respectively. H atoms were positioned geometrically, with C—H = 0.93, 0.96 and 0.97 Å for aromatic, methyl and methylene H atoms, respectively, and constrained to ride on their parent atoms, with *U*_iso_(H) = *xU*_eq_(C) where *x* = 1.5 for methyl H and *x* = 1.2 for all other H atoms.

### Crystal data

**Table:**

[Sn(CH_3_)_3_(C_6_H_5_O_2_S)]	*Z* = 4
*M**_r_* = 304.95	*F*(000) = 600
Triclinic, *P*1	*D*_x_ = 1.652 Mg m^−^^3^
Hall symbol: -P 1	Mo *K*α radiation, λ = 0.71073 Å
*a* = 10.0677 (9) Å	Cell parameters from 2369 reflections
*b* = 10.839 (1) Å	θ = 2.5–25.0°
*c* = 13.2024 (17) Å	µ = 2.23 mm^−^^1^
α = 107.813 (2)°	*T* = 298 K
β = 105.606 (1)°	Block, colorless
γ = 105.030 (1)°	0.55 × 0.50 × 0.48 mm
*V* = 1226.0 (2) Å^3^	

### Data collection

**Table:**

Bruker SMART CCD area-detector diffractometer	4227 independent reflections
Radiation source: fine-focus sealed tube	2865 reflections with *I* > 2σ(*I*)
graphite	*R*_int_ = 0.039
φ and ω scans	θ_max_ = 25.0°, θ_min_ = 2.1°
Absorption correction: multi-scan (*SADABS*; Sheldrick, 1996)	*h* = −11→11
*T*_min_ = 0.374, *T*_max_ = 0.415	*k* = −11→12
6334 measured reflections	*l* = −15→15

### Refinement

**Table:**

Refinement on *F*^2^	Primary atom site location: structure-invariant direct methods
Least-squares matrix: full	Secondary atom site location: difference Fourier map
*R*[*F*^2^ > 2σ(*F*^2^)] = 0.046	Hydrogen site location: inferred from neighbouring sites
*wR*(*F*^2^) = 0.143	H-atom parameters constrained
*S* = 0.97	*w* = 1/[σ^2^(*F*_o_^2^) + (0.0669*P*)^2^ + 2.6141*P*] where *P* = (*F*_o_^2^ + 2*F*_c_^2^)/3
4227 reflections	(Δ/σ)_max_ = 0.048
282 parameters	Δρ_max_ = 0.98 e Å^−^^3^
0 restraints	Δρ_min_ = −0.94 e Å^−^^3^

### Special details

**Table:**

Experimental. Yield 76%; m.p. 458 (3) K. Analysis calculated (%) for C_9_H_14_O_2_S_1_Sn_1_ (Mr = 304.95): C, 35.42; H, 4.59. Found: C, 35.53; H, 4.67.
Geometry. All e.s.d.'s (except the e.s.d. in the dihedral angle between two l.s. planes) are estimated using the full covariance matrix. The cell e.s.d.'s are taken into account individually in the estimation of e.s.d.'s in distances, angles and torsion angles; correlations between e.s.d.'s in cell parameters are only used when they are defined by crystal symmetry. An approximate (isotropic) treatment of cell e.s.d.'s is used for estimating e.s.d.'s involving l.s. planes.
Refinement. Refinement of *F*^2^ against ALL reflections. The weighted *R*-factor *wR* and goodness of fit *S* are based on *F*^2^, conventional *R*-factors *R* are based on *F*, with *F* set to zero for negative *F*^2^. The threshold expression of *F*^2^ > σ(*F*^2^) is used only for calculating *R*-factors(gt) *etc*. and is not relevant to the choice of reflections for refinement. *R*-factors based on *F*^2^ are statistically about twice as large as those based on *F*, and *R*- factors based on ALL data will be even larger.

### Fractional atomic coordinates and isotropic or equivalent isotropic displacement parameters (Å^2^)

**Table:**

	*x*	*y*	*z*	*U*_iso_*/*U*_eq_	Occ. (<1)
Sn1	0.00471 (6)	0.03382 (5)	0.21785 (4)	0.0461 (2)	
Sn2	−0.41279 (6)	0.16957 (6)	0.33911 (5)	0.0545 (2)	
O1	−0.1169 (7)	0.1535 (7)	0.3189 (5)	0.0680 (15)	
O2	−0.1760 (6)	0.2957 (6)	0.4423 (5)	0.0676 (14)	
O3	0.0962 (6)	−0.0894 (6)	0.1133 (5)	0.0606 (13)	
O4	0.3251 (7)	0.0451 (7)	0.2369 (6)	0.0690 (15)	
S1	0.2843 (5)	0.3983 (5)	0.8011 (4)	0.1006 (13)	0.799 (9)
S2	0.3209 (8)	−0.4916 (6)	0.1179 (6)	0.119 (2)	0.618 (7)
C6'	0.247 (7)	0.323 (7)	0.756 (4)	0.1006 (13)	0.201 (9)
H6'	0.2871	0.2683	0.7866	0.121*	0.201 (9)
C12'	0.385 (3)	−0.442 (4)	0.161 (3)	0.100 (3)	0.382 (7)
H12'	0.4562	−0.4806	0.1770	0.120*	0.382 (7)
C1	−0.0805 (10)	0.2573 (10)	0.4090 (8)	0.0614 (17)	
C2	0.0805 (10)	0.3498 (10)	0.4811 (8)	0.0670 (18)	
H2A	0.1420	0.3084	0.4483	0.080*	
H2B	0.0979	0.4395	0.4758	0.080*	
C3	0.2552 (11)	0.3558 (12)	0.6612 (8)	0.088 (2)	
H3	0.3158	0.3241	0.6265	0.106*	
C4	0.1300 (10)	0.3741 (10)	0.6046 (8)	0.0668 (18)	
C5	0.0582 (11)	0.4211 (11)	0.6774 (7)	0.083 (2)	
H5	−0.0335	0.4284	0.6505	0.100*	
C6	0.1366 (18)	0.4563 (19)	0.7945 (10)	0.092 (2)	0.799 (9)
H6	0.1142	0.5015	0.8562	0.111*	0.799 (9)
S1'	0.142 (2)	0.4122 (19)	0.7986 (12)	0.092 (2)	0.201 (9)
C7	0.2353 (10)	−0.0589 (9)	0.1473 (8)	0.0613 (17)	
C8	0.2889 (11)	−0.1548 (10)	0.0747 (9)	0.0717 (19)	
H8A	0.2320	−0.1808	−0.0057	0.086*	
H8B	0.3922	−0.1054	0.0902	0.086*	
C9	0.3697 (14)	−0.3484 (11)	0.0930 (10)	0.097 (2)	
H9	0.4549	−0.3174	0.0784	0.117*	
C10	0.2751 (12)	−0.2818 (11)	0.0960 (10)	0.0784 (19)	
C11	0.141 (2)	−0.3678 (19)	0.0865 (18)	0.092 (3)	0.618 (7)
H11	0.0564	−0.3457	0.0747	0.111*	0.618 (7)
C12	0.1449 (17)	−0.5016 (18)	0.0973 (18)	0.087 (3)	0.618 (7)
H12	0.0672	−0.5738	0.0935	0.105*	0.618 (7)
C11'	0.216 (3)	−0.312 (2)	0.175 (3)	0.085 (3)	0.382 (7)
H11'	0.1699	−0.2589	0.2123	0.102*	0.382 (7)
S2'	0.2352 (12)	−0.4508 (9)	0.1964 (10)	0.112 (2)	0.382 (7)
C13	0.0846 (11)	−0.0140 (10)	0.3604 (8)	0.068 (2)	
H13A	0.1823	0.0536	0.4104	0.102*	
H13B	0.0887	−0.1054	0.3341	0.102*	
H13C	0.0191	−0.0120	0.4015	0.102*	
C14	−0.2133 (10)	−0.0952 (9)	0.0935 (8)	0.066 (2)	
H14A	−0.2219	−0.1910	0.0666	0.098*	
H14B	−0.2317	−0.0679	0.0300	0.098*	
H14C	−0.2848	−0.0850	0.1279	0.098*	
C15	0.1068 (11)	0.2206 (9)	0.2013 (8)	0.069 (2)	
H15A	0.0632	0.2869	0.2275	0.104*	
H15B	0.0919	0.2003	0.1221	0.104*	
H15C	0.2113	0.2589	0.2468	0.104*	
C16	−0.4000 (12)	−0.0100 (11)	0.3667 (9)	0.082 (3)	
H16A	−0.3111	0.0163	0.4316	0.123*	
H16B	−0.3980	−0.0755	0.2997	0.123*	
H16C	−0.4851	−0.0521	0.3815	0.123*	
C17	−0.4101 (11)	0.2078 (11)	0.1919 (8)	0.075 (3)	
H17A	−0.4097	0.1274	0.1355	0.113*	
H17B	−0.3227	0.2871	0.2128	0.113*	
H17C	−0.4969	0.2263	0.1605	0.113*	
C18	−0.4695 (12)	0.3080 (12)	0.4558 (10)	0.099 (4)	
H18A	−0.5723	0.2659	0.4429	0.149*	
H18B	−0.4530	0.3933	0.4445	0.149*	
H18C	−0.4090	0.3278	0.5330	0.149*	

### Atomic displacement parameters (Å^2^)

**Table:**

	*U*^11^	*U*^22^	*U*^33^	*U*^12^	*U*^13^	*U*^23^
Sn1	0.0353 (3)	0.0473 (3)	0.0463 (3)	0.0115 (2)	0.0107 (2)	0.0144 (3)
Sn2	0.0379 (3)	0.0577 (4)	0.0552 (4)	0.0155 (3)	0.0156 (3)	0.0103 (3)
O1	0.045 (3)	0.069 (3)	0.067 (3)	0.018 (2)	0.015 (2)	0.007 (2)
O2	0.045 (3)	0.071 (3)	0.069 (3)	0.024 (2)	0.018 (2)	0.007 (2)
O3	0.050 (3)	0.058 (3)	0.070 (3)	0.023 (2)	0.025 (2)	0.017 (2)
O4	0.049 (3)	0.065 (3)	0.081 (3)	0.021 (2)	0.025 (2)	0.015 (3)
S1	0.083 (2)	0.112 (3)	0.085 (2)	0.0325 (19)	0.0064 (18)	0.0385 (19)
S2	0.105 (3)	0.081 (3)	0.136 (4)	0.030 (3)	0.018 (3)	0.025 (3)
C6'	0.083 (2)	0.112 (3)	0.085 (2)	0.0325 (19)	0.0064 (18)	0.0385 (19)
C12'	0.086 (5)	0.072 (4)	0.116 (5)	0.025 (4)	0.022 (4)	0.025 (4)
C1	0.045 (3)	0.068 (3)	0.064 (3)	0.022 (3)	0.019 (3)	0.017 (3)
C2	0.048 (3)	0.078 (3)	0.069 (3)	0.026 (3)	0.023 (3)	0.019 (3)
C3	0.069 (3)	0.096 (4)	0.081 (3)	0.032 (3)	0.012 (3)	0.026 (3)
C4	0.055 (3)	0.077 (3)	0.071 (3)	0.028 (3)	0.021 (3)	0.031 (3)
C5	0.068 (3)	0.092 (3)	0.076 (3)	0.029 (3)	0.015 (3)	0.028 (3)
C6	0.078 (3)	0.099 (4)	0.084 (3)	0.025 (3)	0.017 (3)	0.035 (3)
S1'	0.078 (3)	0.099 (4)	0.084 (3)	0.025 (3)	0.017 (3)	0.035 (3)
C7	0.055 (3)	0.057 (3)	0.077 (3)	0.023 (3)	0.032 (3)	0.026 (3)
C8	0.062 (3)	0.065 (3)	0.087 (4)	0.023 (3)	0.035 (3)	0.024 (3)
C9	0.082 (4)	0.076 (4)	0.110 (4)	0.023 (3)	0.027 (3)	0.023 (3)
C10	0.068 (3)	0.065 (3)	0.099 (3)	0.028 (3)	0.032 (3)	0.026 (3)
C11	0.080 (4)	0.073 (4)	0.107 (4)	0.023 (3)	0.029 (4)	0.025 (3)
C12	0.081 (4)	0.065 (4)	0.109 (4)	0.025 (4)	0.031 (4)	0.031 (4)
C11'	0.075 (4)	0.067 (4)	0.101 (4)	0.024 (3)	0.030 (3)	0.024 (3)
S2'	0.105 (4)	0.078 (4)	0.127 (4)	0.020 (3)	0.028 (4)	0.033 (3)
C13	0.063 (4)	0.075 (5)	0.058 (4)	0.019 (4)	0.015 (4)	0.028 (4)
C14	0.045 (4)	0.064 (5)	0.064 (5)	0.007 (4)	0.006 (4)	0.018 (4)
C15	0.065 (5)	0.062 (4)	0.074 (5)	0.017 (4)	0.021 (4)	0.030 (4)
C16	0.069 (5)	0.079 (5)	0.079 (5)	0.017 (4)	0.011 (4)	0.033 (4)
C17	0.058 (5)	0.078 (5)	0.070 (5)	0.010 (4)	0.011 (4)	0.029 (4)
C18	0.064 (6)	0.096 (6)	0.093 (6)	0.027 (5)	0.030 (5)	−0.016 (5)

### Geometric parameters (Å, °)

**Table:**

Sn1—C13	2.111 (8)	C5—H5	0.9300
Sn1—C15	2.125 (9)	C6—H6	0.9300
Sn1—C14	2.129 (8)	C7—C8	1.499 (12)
Sn1—O3	2.176 (5)	C8—C10	1.467 (13)
Sn1—O1	2.368 (6)	C8—H8A	0.9700
Sn2—C17	2.113 (9)	C8—H8B	0.9700
Sn2—C18	2.110 (9)	C9—C10	1.337 (14)
Sn2—C16	2.116 (10)	C9—H9	0.9300
Sn2—O2	2.198 (6)	C10—C11	1.38 (2)
Sn2—O4^i^	2.394 (6)	C10—C11'	1.42 (3)
O1—C1	1.243 (10)	C11—C12	1.51 (2)
O2—C1	1.271 (10)	C11—H11	0.9300
O3—C7	1.266 (10)	C12—H12	0.9300
O4—C7	1.252 (11)	C11'—S2'	1.662 (15)
O4—Sn2^ii^	2.394 (6)	C11'—H11'	0.9300
S1—C3	1.682 (10)	C13—H13A	0.9600
S1—C6	1.751 (14)	C13—H13B	0.9600
S2—C9	1.662 (11)	C13—H13C	0.9600
S2—C12	1.688 (13)	C14—H14A	0.9600
C6'—C3	1.420 (10)	C14—H14B	0.9600
C6'—S1'	1.690 (17)	C14—H14C	0.9600
C6'—H6'	0.9300	C15—H15A	0.9600
C12'—C9	1.55 (4)	C15—H15B	0.9600
C12'—S2'	1.688 (16)	C15—H15C	0.9600
C12'—H12'	0.9300	C16—H16A	0.9600
C1—C2	1.511 (12)	C16—H16B	0.9600
C2—C4	1.487 (12)	C16—H16C	0.9600
C2—H2A	0.9700	C17—H17A	0.9600
C2—H2B	0.9700	C17—H17B	0.9600
C3—C4	1.378 (8)	C17—H17C	0.9600
C3—H3	0.9300	C18—H18A	0.9600
C4—C5	1.403 (8)	C18—H18B	0.9600
C5—C6	1.409 (9)	C18—H18C	0.9600
C5—S1'	1.642 (14)		
			
C13—Sn1—C15	125.3 (4)	C10—C8—H8A	109.0
C13—Sn1—C14	118.1 (4)	C7—C8—H8A	109.0
C15—Sn1—C14	115.6 (4)	C10—C8—H8B	109.0
C13—Sn1—O3	94.8 (3)	C7—C8—H8B	109.0
C15—Sn1—O3	94.7 (3)	H8A—C8—H8B	107.8
C14—Sn1—O3	89.8 (3)	C10—C9—C12'	121.4 (13)
C13—Sn1—O1	87.5 (3)	C10—C9—S2	113.8 (10)
C15—Sn1—O1	87.8 (3)	C10—C9—H9	123.1
C14—Sn1—O1	84.9 (3)	C12'—C9—H9	111.0
O3—Sn1—O1	174.7 (2)	S2—C9—H9	123.1
C17—Sn2—C18	115.9 (5)	C9—C10—C11	110.8 (12)
C17—Sn2—C16	126.4 (4)	C9—C10—C11'	103.9 (13)
C18—Sn2—C16	116.9 (5)	C11—C10—C11'	45.4 (13)
C17—Sn2—O2	95.0 (3)	C9—C10—C8	125.4 (10)
C18—Sn2—O2	89.8 (3)	C11—C10—C8	121.2 (12)
C16—Sn2—O2	94.0 (3)	C11'—C10—C8	123.7 (11)
C17—Sn2—O4^i^	86.8 (3)	C10—C11—C12	113.3 (16)
C18—Sn2—O4^i^	85.8 (3)	C10—C11—H11	123.4
C16—Sn2—O4^i^	88.2 (3)	C12—C11—H11	123.3
O2—Sn2—O4^i^	175.6 (2)	C11—C12—S2	105.0 (12)
C1—O1—Sn1	137.2 (6)	C11—C12—H12	127.5
C1—O2—Sn2	118.4 (6)	S2—C12—H12	127.5
C7—O3—Sn1	119.8 (6)	C10—C11'—S2'	113.7 (18)
C7—O4—Sn2^ii^	139.9 (6)	C10—C11'—H11'	123.2
C3—S1—C6	94.4 (6)	S2'—C11'—H11'	123.2
C9—S2—C12	94.6 (8)	C11'—S2'—C12'	95.9 (18)
C3—C6'—S1'	100.4 (16)	Sn1—C13—H13A	109.5
C3—C6'—H6'	129.8	Sn1—C13—H13B	109.5
S1'—C6'—H6'	129.8	H13A—C13—H13B	109.5
C9—C12'—S2'	99.0 (18)	Sn1—C13—H13C	109.5
C9—C12'—H12'	130.5	H13A—C13—H13C	109.5
S2'—C12'—H12'	130.5	H13B—C13—H13C	109.5
O1—C1—O2	122.2 (8)	Sn1—C14—H14A	109.5
O1—C1—C2	121.5 (8)	Sn1—C14—H14B	109.5
O2—C1—C2	116.2 (8)	H14A—C14—H14B	109.5
C4—C2—C1	115.5 (8)	Sn1—C14—H14C	109.5
C4—C2—H2A	108.4	H14A—C14—H14C	109.5
C1—C2—H2A	108.4	H14B—C14—H14C	109.5
C4—C2—H2B	108.4	Sn1—C15—H15A	109.5
C1—C2—H2B	108.4	Sn1—C15—H15B	109.5
H2A—C2—H2B	107.5	H15A—C15—H15B	109.5
C4—C3—C6'	113 (2)	Sn1—C15—H15C	109.5
C4—C3—S1	111.4 (8)	H15A—C15—H15C	109.5
C4—C3—H3	124.3	H15B—C15—H15C	109.5
C6'—C3—H3	116.6	Sn2—C16—H16A	109.5
S1—C3—H3	124.3	Sn2—C16—H16B	109.5
C3—C4—C5	112.4 (9)	H16A—C16—H16B	109.5
C3—C4—C2	123.1 (8)	Sn2—C16—H16C	109.5
C5—C4—C2	124.5 (8)	H16A—C16—H16C	109.5
C4—C5—C6	114.5 (10)	H16B—C16—H16C	109.5
C4—C5—S1'	105.5 (10)	Sn2—C17—H17A	109.5
C4—C5—H5	122.7	Sn2—C17—H17B	109.5
C6—C5—H5	122.7	H17A—C17—H17B	109.5
S1'—C5—H5	129.4	Sn2—C17—H17C	109.5
C5—C6—S1	106.5 (9)	H17A—C17—H17C	109.5
C5—C6—H6	126.8	H17B—C17—H17C	109.5
S1—C6—H6	126.8	Sn2—C18—H18A	109.5
C5—S1'—C6'	99.5 (15)	Sn2—C18—H18B	109.5
O4—C7—O3	122.5 (8)	H18A—C18—H18B	109.5
O4—C7—C8	120.9 (8)	Sn2—C18—H18C	109.5
O3—C7—C8	116.5 (8)	H18A—C18—H18C	109.5
C10—C8—C7	112.8 (8)	H18B—C18—H18C	109.5

Symmetry codes: (i) *x*−1, *y*, *z*; (ii) *x*+1, *y*, *z*.

## References

[bb1] Addison, A. W., Rao, T. N., Reedijk, J., Rijn, J. V. & Verschoor, G. C. (1984). *J. Chem. Soc. Dalton Trans.****2***, 1349–1356.

[bb2] Ma, C., Li, J., Zhang, R. & Wang, D. (2006). *J. Organomet. Chem.* **691**, 1713–1721.

[bb3] Sheldrick, G. M. (1996). *SADABS* University of Göttingen, Germany.

[bb4] Sheldrick, G. M. (2008). *Acta Cryst.* A**64**, 112–122. 10.1107/S0108767307043930 18156677

[bb5] Siemens (1996). *SMART* and *SAINT* Siemens Analytical X-ray Instruments Inc., Madison, Wisconsin, USA.

